# Plastic Change along the Intact Crossed Pathway in Acute Phase of Cerebral Ischemia Revealed by Optical Intrinsic Signal Imaging

**DOI:** 10.1155/2016/1923160

**Published:** 2016-04-06

**Authors:** Xiaoli Guo, Yongzhi He, Hongyang Lu, Yao Li, Xin Su, Ying Jiang, Shanbao Tong

**Affiliations:** School of Biomedical Engineering, Shanghai Jiao Tong University, Shanghai 200240, China

## Abstract

The intact crossed pathway via which the contralesional hemisphere responds to the ipsilesional somatosensory input has shown to be affected by unilateral stroke. The aim of this study was to investigate the plasticity of the intact crossed pathway in response to different intensities of stimulation in a rodent photothrombotic stroke model. Using optical intrinsic signal imaging, an overall increase of the contralesional cortical response was observed in the acute phase (≤48 hours) after stroke. In particular, the contralesional hyperactivation is more prominent under weak stimulations, while a strong stimulation would even elicit a depressed response. The results suggest a distinct stimulation-response pattern along the intact crossed pathway after stroke. We speculate that the contralesional hyperactivation under weak stimulations was due to the reorganization for compensatory response to the weak ipsilateral somatosensory input.

## 1. Introduction

The sensory inputs to brain are mainly integrated and processed in the contralateral hemisphere along the crossed pathway via the corpus callosum [1, 2]. Meanwhile, the sensory signals would also be transmitted to the ipsilateral cortex through the uncrossed pathway, though such an ipsilateral connection is usually suppressed due to the interhemispheric inhibition (IHI) in healthy subjects [3]. However, when the brain suffers an ischemic stroke, both afferent and efferent pathways will be injured and thus lead to malfunction in motor and/or sensory systems. In response to such an ischemic lesion, the brain undergoes a battery of changes, referred to as plasticity, in both injured and intact hemispheres to retain the function along both contralateral and ipsilateral connections [4, 5].

So far, many researchers have focused on the bihemispheric changes in response to stimulation of the stroke-affected body side. Enlarged receptive field in the periinfarct area has been observed after injury [6–8]. Cortical remapping accompanied by new structural connections with periinfarct zone was also found in the recovery phase after stroke [9]. On the other hand, plastic changes in the contralesional cortex have also been reported but with inconsistent results. Neuroimaging studies by either ^18^F-fluorodeoxyglucose small-animal positron emission tomography (FDG micro-PET) or optical intrinsic signal (OIS) on rats did not show metabolic alteration in the contralesional hemisphere [10, 11]. However, another three animal studies using functional magnetic resonance imaging (fMRI), [^14^C]-2-doexyglucose (2DG) autoradiography, or voltage-sensitive dye imaging found a significant increase of ipsilateral response in the contralesional hemisphere [12–14]. Nevertheless, clinical reports did show that the contralesional uncrossed pathway related to the recovery after stroke [15–18].

Besides the direct bihemispheric effects on the affected body side, the intact crossed pathway from ipsilesional body to the contralesional homotopic cortex plays a special role in stroke rehabilitation due to the modulation of IHI [19]. Deafferentation of the intact hand could improve sensory and motor deficits of the affected hand [20, 21]. Clinically, constraint-induced movement therapy which combines restraint of the intact limb and intensive use of the affected limb is now widely used in therapy of patients with hemiparetic stroke [22]. Therefore, it is important to understand the nature of the intact crossed pathway after stroke. Unexpectedly, the “intact” crossed pathway also changed after unilateral stroke, producing subtle deficits in the ipsilesional “less-affected” body side [23]. Enhanced responses in the contralesional cortex to tactile stimulation of the ipsilesional forelimb were observed in hyperacute phase (<2 h) of stroke in aspect of either peak response or activated area [12]. However, whether such a hyperactive response along the intact crossed pathway depends on specific nature of stimulation (e.g., intensity, frequency, and duration) remains unknown. In this study, using OIS imaging, plastic changes along the intact crossed pathway in response to stimulation at different intensities in acute phase of stroke (≤48 h) were investigated in a rodent photothrombotic stroke model.

## 2. Materials and Methods

### 2.1. Animal Preparation

The experimental protocol was approved by the institutional animal care and use committee of Med-X Research Institute, Shanghai Jiao Tong University. Twenty-eight male Sprague-Dawley (SD) rats (350 ± 50 g, 14 wk, Slac Laboratory Animal, Shanghai, China) were used in this study. During the experiment, all rats were anesthetized with isoflurane (5% initial, 2% for maintenance; mixed in the air) and were constrained in a stereotaxic frame (Benchmark Deluxe*™*, MyNeurolab.com, St. Louis, MO, USA). Rectal temperature was monitored and maintained at 37.0 ± 0.2°C with a heating pad connected to a DC temperature control module (FHC Inc., Bowdoin, ME, USA). Surgical procedures were operated with sterile caution.

Cranial windows were carefully prepared 12 hours before the photothrombotic stroke or sham surgery. First, the scalp was removed to expose the skull. Then two cranial windows, 3 mm × 4 mm each over either hemisphere, were created by thinning the skull with a high speed dental drill (Fine Science Tools Inc., North Vancouver, Canada). Finally, dental cement was used to form an imaging chamber enclosing the cranial windows.

### 2.2. Photothrombotic Stroke Model

Stroke was induced by means of photoactivation of the preinjected Rose Bengal, which is specifically responsive to 532 nm laser illumination, so as to produce damage to endothelial cell membranes. Such photoactivated damage could result in platelet aggregation and vascular thrombosis [24, 25].

**Figure 1:**
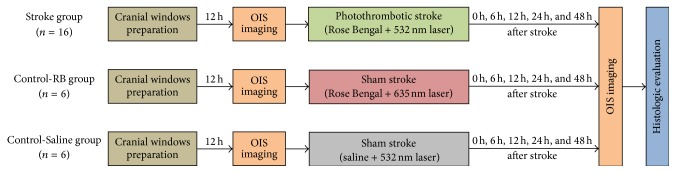
Experimental protocols. In Stroke group, the cranial imaging windows were prepared 12 h before the stroke surgery. Optical intrinsic signal (OIS) images were recorded for assessing the somatosensory response at different stages of the experiment (baseline before stroke, and 0 h, 6 h, 12 h, 24 h, and 48 h after stroke). Histologic analysis was performed to confirm the lesion after the experiment. Different from Stroke group, the two Control groups underwent sham surgery instead of photothrombotic stroke. Considering the potential influence of the Rose Bengal or laser illumination, we included two Control groups (Control-RB and Control-Saline). Control-RB rats were illuminated by nonresponsive red laser (635 ± 5 nm, 20 mW), while Control-Saline rats were injected with the same dosage of saline.

After preparation of the cranial windows, rats were randomly assigned to three groups (Figure 1), that is, one Stroke group (Stroke, *n* = 16) and two Control groups (Control-RB, *n* = 6; Control-Saline, *n* = 6; see the details below).

**Figure 2:**
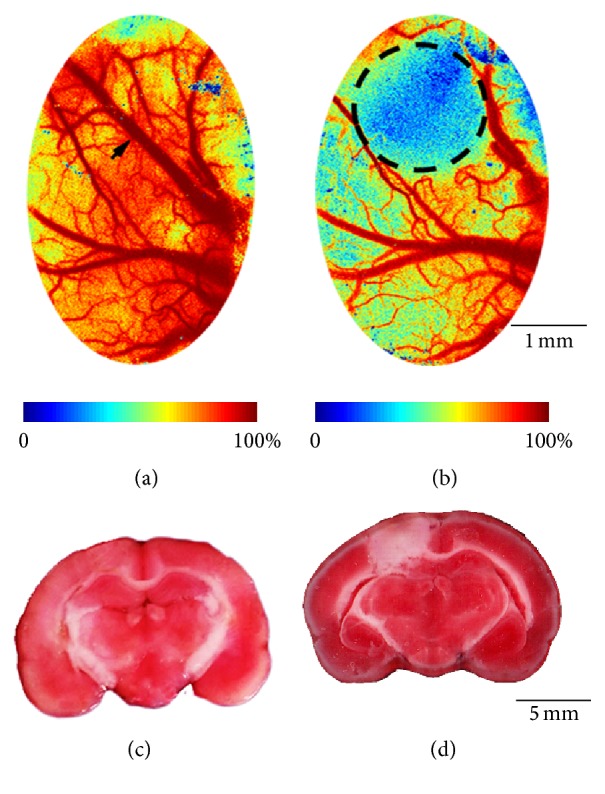
Laser speckle imaging (LSI) and triphenyltetrazolium chloride (TTC) staining results of photothrombosis. LSI images before (a) and after (b) photothrombotic stroke. The flow in the vessel (see the arrow area) apparently disappeared immediately after the thrombi formed, indicating the success of stroke model. The color map stands for the relative speed of blood flow, and the circle shows the main affected area due to ischemia. Representative TTC stained coronal sections of brain slices in Control group (c) and Stroke group (d). Unstained area depicts infarcted S1HL after photothrombotic stroke.

For the rats in Stroke group, Rose Bengal (80 mg/kg) was injected through tail vein two minutes before the illumination of a green laser (532 ± 5 nm, 20 mW, and 15 min). The beam was focused on an area (2 mm diameter) in the left primary somatosensory cortex for hindlimb (S1HL, 2 mm lateral, and 1 mm posterior to bregma) [26–28]. The success of stroke model was then confirmed by real-time laser speckle imaging (LSI) (Figures 2(a) and 2(b)) [29]. Rats in Control groups underwent all the above procedures except that Control-RB rats were illuminated by a nonresponsive red laser (635 ± 5 nm, 20 mW), while Control-Saline rats were injected with the same dosage of saline instead of Rose Bengal before the green laser illumination.

### 2.3. OIS Imaging and Processing

**Figure 3:**
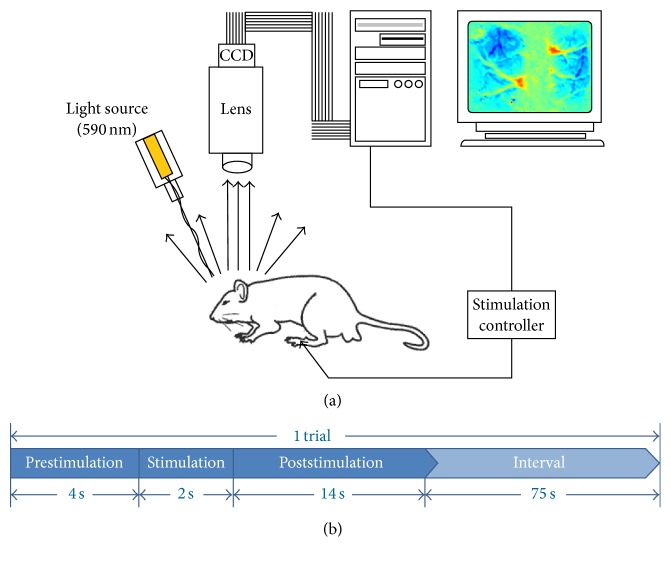
Schematic of optical intrinsic signal (OIS) imaging system and hindlimb electrical stimulation protocol. (a) The head of rat was fixed in a stereotaxic frame and then illuminated by a 590 nm LED throughout the experiment. The ipsilesional hindlimb was stimulated with different intensity of currents (2 mA, 3.5 mA, and 5 mA). OIS images were captured by a CCD camera connected with a lens and then transferred to the computer for further processing. (b) Each trial of OIS recordings consisted of a 4 s resting state and a 2 s electrical stimulation followed by a 14 s poststimulation state. For each intensity of stimulation, 12 trials of data were recorded at an intertrial interval of 75 s.

In order to get the OIS images, anesthetized rats were placed on an imaging platform (Figure 3(a)). Cranial windows were filled with mineral oil and illuminated by a monochromatic light source (590 nm, Thorlabs, Newton, NJ, USA). OIS images were acquired at 40 fps by a 12-bit CCD (640 (W) × 480 (H), acA2040-180 km, Basler, German) connected with a camera lens (AF-S Micro 105 mm f/2.8 G IF-ED VR, Nikkor, Japan). OIS imaging was performed at six time points, that is, baseline before stroke and 0 h, 6 h, 12 h, 24 h, and 48 h after stroke. At each time point, three blocks of OIS images were recorded sequentially at the intensity of 2 mA, 3.5 mA, and 5 mA stimulation (rectangular constant current pulses, 0.3 ms, 5 Hz), respectively. There was a 2 min interval between two consequential blocks. For each intensity of stimulation, twelve trials of OIS data were recorded at an intertrial interval of 75 s. Each trial of OIS recording consisted of a 4 s resting state plus a 2 s electrical stimulation followed by a 14 s poststimulation state (Figure 3(b)). Electrical stimulation was carried out by a programmable stimulator (YC-2, Cheng Yi, China) using two needle electrodes subcutaneously inserted into the left hind paw. The left hind toes were observed twitching due to electrical stimulation at 2 mA, 3.5 mA, or 5 mA. Before OIS imaging, LSI images were collected by a laser source (785 nm, Thorlabs, Newton, NJ, USA) for vessel segmentation. All imaging procedures were programed by MATLAB (Ver. 2012a, MathWorks, Natick, MA, USA).

OIS data were off-line analyzed using customized software. To quantify the relative change of cerebral blood volume caused by the stimulation, response intensity at each pixel was normalized by the average of prestimulation baseline. Then, every 20 continuous frames were combined to form a 0.5 s resolution OIS profile and then averaged over all trials by stimulation intensity to improve the signal-to-noise ratio [30]. To avoid the interference from large vessels, we removed the major arteries and veins using LSI-based blood vessel segmentation (see Figure  2 in [31]). Finally, OIS data were filtered by a spatial 2D Gaussian filter (*σ* = 3) to suppress the background noises.

At each intensity of stimulation, maximum response was defined as the mean value of ten pixels in the frame with the strongest response (i.e., 3 s after stimulation onset). In this frame, region with more than 50% maximum response was defined as the response area [11]. The total OIS value within the response area was used as the absolute response index to stimulation. In order to eliminate the influence of variance across subjects, the responses to 3.5 mA and 5 mA stimulations were normalized by that to 2 mA stimulation, or called relative response, to study the response-stimulation relation.

### 2.4. Histology

In order to confirm the success of stroke, six rats in Stroke group and one rat in each Control group were selected for histological analysis after OIS imaging at 48 h after stroke. The brains were carefully removed and sectioned into 3 mm slices along the coronal plane. The tissues were then incubated in 4% triphenyltetrazolium chloride (TTC) at 37°C for 10 min in the dark [32]. The TTC stained sections were scanned for analyzing the lesion. The total infarct volume was determined by the difference of the intact volumes between two hemispheres using ImageJ software (National Institutes of Health, Bethesda, Maryland) [33].

### 2.5. Statistical Analysis

The responses were statistically analyzed with repeated measures analysis of variance (ANOVA) using the Greenhouse-Geisser correction. At first, the absolute response index was compared between two Control groups by a three-way repeated measures ANOVA, taking GROUP (Control-RB versus Control-Saline) as the between-subjects factor and STAGE (baseline, 0 h, 6 h, 12 h, 24 h and 48 h after stroke) and INTENSITY (2 mA, 3.5 mA and 5 mA) as the within-subjects factors. As no significant difference between Control-RB and Control-Saline was found; therefore, two Control groups were merged into one Control group in the following analysis (see the results). The response pattern over time in Control group was assessed by a two-way (STAGE × INTENSITY) repeated measures ANOVA. Then, we compared Stroke group with Control group by a three-way (GROUP × STAGE × INTENSITY) repeated measures ANOVA, in which the between-subjects factor GROUP had two levels (Control versus Stroke). At last, two separated two-way (STAGE × INTENSITY) repeated measures ANOVAs were performed on the absolute and relative response indices of Stroke group to investigate the response change after stroke.

## 3. Results

### 3.1. Infarct Volume of Photothrombotic Stroke

Histological results were analyzed to confirm the success and stability of the photothrombotic stroke. The mean (±SD) infarct volume was 41.45 ± 3.74 mm^3^ with a coefficient of variation (CV, standard deviation/mean) of 9.02%. Meanwhile, none of the rats in Control group exhibited an infarct. The representative histological images were shown in Figures 2(c) and 2(d).

### 3.2. Response Pattern along the Intact Crossed Pathway in Control Groups

Contralesional (right) cortical responses to ipsilesional (left) hindlimb stimulation were similar in Control-RB group and Control-Saline group. When comparing between two Control groups, we did not find significant main effect of GROUP (Control-RB versus Control-Saline, *F*(1,10) = 2.406, *p* = 0.152), or significant interactions GROUP × STAGE (*F*(5,50) = 1.448, *p* = 0.237), or GROUP × INTENSITY (*F*(2,20) = 1.976, *p* = 0.189). Therefore, we merged Control-RB group and Control-Saline group into a single Control group in the following analyses.

**Figure 4:**
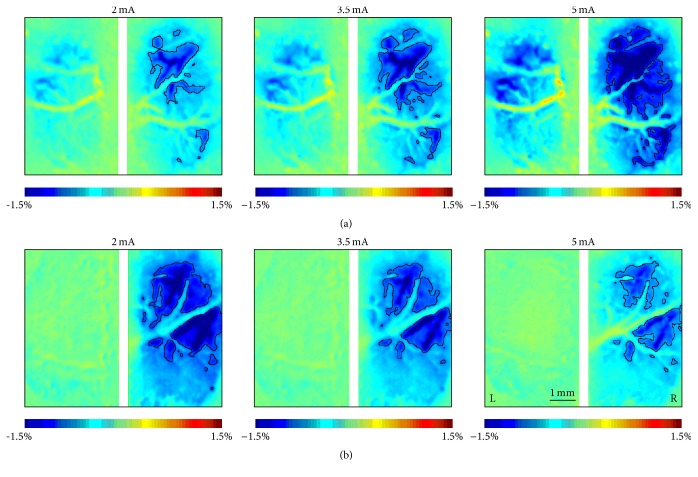
Optical intrinsic signal (OIS) images at 24 h after sham surgery or photothrombotic stroke in Control group (a) and Stroke group (b). During the imaging, the left hind paw was stimulated with different intensity of currents at 2 mA, 3.5 mA, and 5 mA, respectively. The contour lines showed the response areas with more than 50% maximum response.

**Figure 5:**
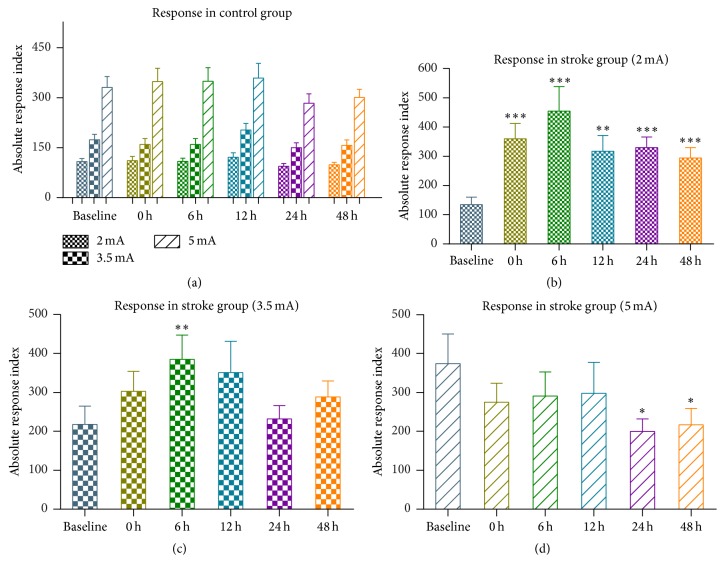
Absolute responses to different stimulation intensities in Control group and Stroke group. (a) Absolute response index in Control group. (b–d) Absolute response index in Stroke group to stimulations at 2 mA, 3.5 mA, and 5 mA, respectively. Error bars represent SEM (^*∗*^ *p* < 0.05; ^*∗∗*^ *p* < 0.01; ^*∗∗∗*^ *p* < 0.001, paired *t*-test compared with baseline).

In Control group (*n* = 12), the cortical response was stable throughout the experiment. The main effect of STAGE (*F*(5, 55) = 1.096, *p* = 0.373) and its interaction with INTENSITY (*F*(10, 110) = 0.984, *p* = 0.427) were insignificant. However, significant main effect of INTENSITY was observed (*F*(2,22) = 175.895, *p* < 0.001). The magnitude of cortical response increased almost linearly with the intensity of stimulation (i.e., 2 mA: 107.03 ± 35.35, 3.5 mA: 167.25 ± 59.37, and 5 mA: 328.74 ± 122.68, Figures 4(a) and 5(a)).

### 3.3. Effect of Photothrombotic Stroke on the Intact Crossed Pathway

The cortical responses along the intact crossed pathway were compared between Stroke group (*n* = 16) and Control group (*n* = 12). Overall, the response in Stroke group (295.51 ± 28.55) was stronger than that in Control group (201.01 ± 32.97), which was due to the significantly enhanced response in Stroke group after ischemic stroke. Significant main effect of GROUP (Stroke versus Control, *F*(1,26) = 4.695, *p* = 0.040) was detected. However, there was no difference between two groups in baseline (*F*(1,26) = 0.399, *p* = 0.533). Interactions GROUP × INTENSITY (*F*(2,52) = 76.595, *p* < 0.001) and GROUP × INTENSITY × STAGE (*F*(10,260) = 3.533, *p* = 0.005) were also significant, indicating distinctly different stimulation-response pattern between two groups. We therefore investigated the stimulation-response pattern of Stroke group at different stages to study the effect of ischemia on the response along the intact crossed pathway.

The effect of stroke on the response along the intact crossed pathway depends on the intensity of stimulation. ANOVA on absolute response index of Stroke group showed significant STAGE × INTENSITY interaction (*F*(10,150) = 5.201, *p* = 0.001). Comparing with the response in the baseline, we found significant increase of response to 2 mA stimulation at any stage after stroke (Figure 5(b), 0 h, 6 h, 12 h, 24 h, and 48 h versus baseline: all *p* < 0.005, paired *t*-test). When responding to 3.5 mA stimulation, poststroke increase was not significant except at 6 h after stroke (Figure 5(c), 6 h versus baseline: *p* = 0.007, paired *t*-test). Conversely, response to 5 mA showed a trend of decrease after stroke and reached the significance level after 24 hours after stroke (Figure 5(d), 24 h versus baseline: *p* = 0.011; 48 h versus baseline: *p* = 0.049, paired *t*-test).

**Figure 6:**
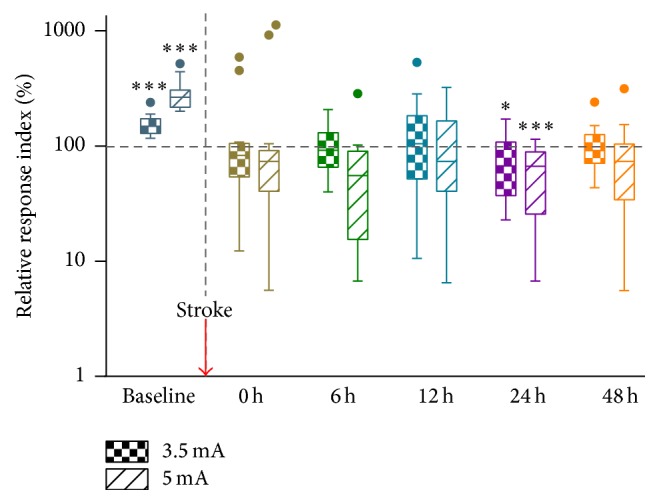
Box-and-whisker plot (Tukey) of relative responses at different time points (baseline, and 0 h, 6 h, 12 h, 24 h, and 48 h after stroke). The relative response is calculated by normalization to the response to 2 mA stimulation. The horizontal dash line stands for the response to 2 mA stimulation, and the vertical dash line represents the time for stroke induction. Error bars represent SEM (^*∗*^ *p* < 0.05; ^*∗∗∗*^ *p* < 0.001, paired *t*-test compared with 2 mA).

The response change after stroke varied at different intensity of stimulation and therefore influenced the corresponding correlation between stimulation intensity and response, which could be revealed by the relative response index. At the baseline, a positive correlation between the stimulation intensity and response was observed in Stroke group; that is, the magnitude of response significantly increased with the stimulation intensity (Figure 6, 2 mA: 100%, 3.5 mA: 154.22 ± 31.19%, and 5 mA: 281.32 ± 87.45%, *F*(2,30) = 49.619, *p* < 0.001), which was similar to Control group. However, such a positive correlation between the stimulation intensity and response disappeared immediately after the ischemic stroke (main effect of INTENSITY: *p* > 0.361 at 0 h, 12 h, and 48 h). In particular, the response significantly decreased as the stimulation intensity increased at 24 h after stroke (Figures 6 and 4(b), 2 mA: 100%, 3.5 mA: 75.14 ± 42.21%, and 5 mA: 60.18 ± 34.75%, *F*(2,30) = 9.771, *p* = 0.001).

## 4. Discussion

### 4.1. Change along the Intact Crossed Pathway after Unilateral Stroke

Our results showed that the intact crossed pathway was affected by unilateral ischemic stroke. Generally, the contralesional cortical response to the ipsilesional limb stimulation in the acute phase (≤48 h) of ischemic stroke was significantly enhanced compared with the baseline level in aspect of either response area [31] or response index. We speculate that such a contralesional hyperactivation could be associated with the disturbance of IHI. In healthy subjects, although both ipsilateral and contralateral somatosensory pathways exist, the brain usually only “feels” the contralateral inputs due to IHI [3], which also can be observed in our OIS images (Figure 4). That is, both ipsilateral (left) and contralateral (right) hemispheres were activated by electrical stimulations of left hind paw; however, the ipsilateral response was much weaker than the contralateral one. After unilateral stroke, the ipsilateral response in the affected (left) hemisphere became weaker and even hard to detect. IHI is therefore disturbed, leading to the hypoactivation in the affected hemisphere and then weakened inhibition to the contralesional cortex. As a result, we observed an enhanced contralesional cortical response, which has also been reported in previous study using voltage-sensitive dye imaging [12].

Note that the contralesional hyperactivation is more prominent when responding to a weak stimulation (e.g., 2 mA), while the brain activation would even be depressed under a strong stimulation (e.g., 5 mA at 24 h), suggesting a distinct stimulation-response pattern along the intact crossed pathway after stroke. One possibility is that the contralesional cortex shifts its responsive preference to weak stimulations during reorganization. In baseline, the brain response was positively correlated with the stimulation intensity. Similarly, a positive relationship was observed between the increases in sensorimotor network activation and the increases in stimulation intensity using fMRI or functional near-infrared spectroscopy (fNIRS) [34, 35]. After unilateral stroke, in order to compensate for damages by ischemia, the contralesional cortex becomes more active to the ipsilateral somatosensory input which is very weak, via the uncrossed pathway [12–14]. Therefore, the contralesional cortical response presented an overall enhancement to weak stimulations from both sides. On the other hand, the response reached saturation more rapidly and even depressed to protect from strong stimulations.

### 4.2. Technical Limitations

OIS measures the stimulation-related change of blood volume relative to prestimulation level. Stimulation elicits changes in blood flow, blood volume, and oxygenation. Changes in the concentration of total hemoglobin (i.e., blood volume), independent of changes in the oxygenation, can be detected by reflectance imaging at 590 nm, since oxyhemoglobin and deoxyhemoglobin have a close absorption coefficient at 590 nm [36]. Our results suggested an abnormal OIS response along the intact crossed pathway after stroke. Nevertheless, more data by other direct and real-time measures, for example, electrophysiological techniques, would help to confirm these findings.

Previous researches have shown that somatosensory stimulation of the affected body side immediately after stroke could be neuroprotective [37–39]. However, whether stimulation of the intact side has an effect on brain injury and/or its recovery remains unknown. Considering the potential influence of prestroke stimulation for baseline OIS imaging, we did a supplementary experiment without prestroke OIS/stimulation (or Stroke-NPS, *n* = 6) and compared them with rats in Stroke group (*n* = 16). No significant main effect of GROUP (Stroke versus Stroke-NPS, *F*(1, 20) = 2.572, *p* = 0.124) or interactions GROUP × STAGE (*F*(4,80) = 0.697, *p* = 0.495) or GROUP × INTENSITY (*F*(2,40) = 1.781, *p* = 0.187) were found. These results implied that the short-time prestroke electrical stimulation had no evident influence on the cortical response after stroke in our experiments. Nevertheless, the present experiments could not exclude the possible influence of poststroke electrical stimulations during OIS imaging, which need to rely on enlarging the sample size.

## References

[1] Sporns O. (2011). *Networks of the Brain*.

[2] Shuler M. G., Krupa D. J., Nicolelis M. A. L. (2001). Bilateral integration of whisker information in the primary somatosensory cortex of rats. *The Journal of Neuroscience*.

[3] Kinsbourne M., Kinsbourne M., Smith W. L. (1974). Mechanisms of hemispheric interaction in man. *Hemisphere Disconnection and Cerebral Function*.

[4] Pascual-Leone A., Amedi A., Fregni F., Merabet L. B. (2005). The plastic human brain cortex. *Annual Review of Neuroscience*.

[5] Murphy T. H., Corbett D. (2009). Plasticity during stroke recovery: from synapse to behaviour. *Nature Reviews Neuroscience*.

[6] Xerri C., Merzenich M. M., Peterson B. E., Jenkins W. (1998). Plasticity of primary somatosensory cortex paralleling sensorimotor skill recovery from stroke in adult monkeys. *Journal of Neurophysiology*.

[7] Coq J. O., Xerri C. (1999). Acute reorganization of the forepaw representation in the rat SI cortex after focal cortical injury: neuroprotective effects of piracetam treatment. *European Journal of Neuroscience*.

[8] Reinecke S., Dinse H. R., Reinke H., Witte O. W. (2003). Induction of bilateral plasticity in sensory cortical maps by small unilateral cortical infarcts in rats. *European Journal of Neuroscience*.

[9] Brown C. E., Aminoltejari K., Erb H., Winship I. R., Murphy T. H. (2009). In vivo voltage-sensitive dye imaging in adult mice reveals that somatosensory maps lost to stroke are replaced over weeks by new structural and functional circuits with prolonged modes of activation within both the peri-infarct zone and distant sites. *The Journal of Neuroscience*.

[10] Carmichael S. T., Tatsukawa K., Katsman D., Tsuyuguchi N., Kornblum H. I. (2004). Evolution of diaschisis in a focal stroke model. *Stroke*.

[11] Johnston D. G., Denizet M., Mostany R., Portera-Cailliau C. (2013). Chronic in vivo imaging shows no evidence of dendritic plasticity or functional remapping in the contralesional cortex after stroke. *Cerebral Cortex*.

[12] Mohajerani M. H., Aminoltejari K., Murphy T. H. (2011). Targeted mini-strokes produce changes in interhemispheric sensory signal processing that are indicative of disinhibition within minutes. *Proceedings of the National Academy of Sciences of the United States of America*.

[13] Dijkhuizen R. M., Ren J., Mandeville J. B. (2001). Functional magnetic resonance imaging of reorganization in rat brain after stroke. *Proceedings of the National Academy of Sciences of the United States of America*.

[14] Jablonka J., Kossut M. (2006). Focal stroke in the barrel cortex of rats enhances ipsilateral response to vibrissal input. *Acta Neurobiologiae Experimentalis*.

[15] Cramer S. C. (2004). Functional imaging in stroke recovery. *Stroke*.

[16] Binkofski F., Seitz R. J. (2004). Modulation of the BOLD-response in early recovery from sensorimotor stroke. *Neurology*.

[17] Feydy A., Carlier R., Roby-Brami A. (2002). Longitudinal study of motor recovery after stroke: recruitment and focusing of brain activation. *Stroke*.

[18] Teasell R., Bayona N. A., Bitensky J. (2005). Plasticity and reorganization of the brain post stroke. *Topics in Stroke Rehabilitation*.

[19] Nowak D. A., Grefkes C., Ameli M., Fink G. R. (2009). Interhemispheric competition after stroke: brain stimulation to enhance recovery of function of the affected hand. *Neurorehabilitation and Neural Repair*.

[20] Werhahn K. J., Mortensen J., Van Boven R. W., Zeuner K. E., Cohen L. G. (2002). Enhanced tactile spatial acuity and cortical processing during acute hand deafferentation. *Nature Neuroscience*.

[21] Voller B., Flöel A., Werhahn K. J., Ravindran S., Wu C. W., Cohen L. G. (2006). Contralateral hand anesthesia transiently improves poststroke sensory deficits. *Annals of Neurology*.

[22] Kwakkel G., Veerbeek J. M., van Wegen E. E. H., Wolf S. L. (2015). Constraint-induced movement therapy after stroke. *The Lancet Neurology*.

[23] Hsu J. E., Jones T. A. (2006). Contralesional neural plasticity and functional changes in the less-affected forelimb after large and small cortical infarcts in rats. *Experimental Neurology*.

[24] Rosenblum W. I., El Sabban F. (1977). Platelet aggregation in the cerebral microcirculation: effect of aspirin and other agents. *Circulation Research*.

[25] Watson B. D., Dietrich W. D., Busto R., Wachtel M. S., Ginsberg M. D. (1985). Induction of reproducible brain infarction by photochemically initiated thrombosis. *Annals of Neurology*.

[26] Ginsberg M. D., Busto R. (1989). Rodent models of cerebral ischemia. *Stroke*.

[27] Zhang S., Murphy T. H. (2007). Imaging the impact of cortical microcirculation on synaptic structure and sensory-evoked hemodynamic responses in vivo. *PLoS Biology*.

[28] Schaffer C. B., Friedman B., Nishimura N. (2006). Two-photon imaging of cortical surface microvessels reveals a robust redistribution in blood flow after vascular occlusion. *PLoS Biology*.

[29] Li Y., Zhu S., Yuan L., Lu H., Li H., Tong S. (2013). Predicting the ischemic infarct volume at the first minute after occlusion in rodent stroke model by laser speckle imaging of cerebral blood flow. *Journal of Biomedical Optics*.

[30] Chen-Bee C. H., Agoncillo T., Xiong Y., Frostig R. D. (2007). The triphasic intrinsic signal: implications for functional imaging. *Journal of Neuroscience*.

[31] He Y., Guo X., Li Y., Lu H., Tong S. Expansion of contralesional sensory representation to ipsilesional hindlimb stimulation in acute phase of ischemic stroke in rat model.

[32] Bederson J. B., Pitts L. H., Tsuji M., Nishimura M. C., Davis R. L., Bartkowski H. (1986). Rat middle cerebral artery occlusion: evaluation of the model and development of a neurologic examination. *Stroke*.

[33] Corbett D., Hamilton M., Colbourne F. (2000). Persistent neuroprotection with prolonged postischemic hypothermia in adult rats subjected to transient middle cerebral artery occlusion. *Experimental Neurology*.

[34] Smith G. V., Alon G., Roys S. R., Gullapalli R. P. (2003). Functional MRI determination of a dose-response relationship to lower extremity neuromuscular electrical stimulation in healthy subjects. *Experimental Brain Research*.

[35] Muthalib M., Re R., Zucchelli L. (2015). Effects of increasing neuromuscular electrical stimulation current intensity on cortical sensorimotor network activation: a time domain fNIRS study. *PLoS ONE*.

[36] Hillman E. M. C. (2007). Optical brain imaging in vivo: techniques and applications from animal to man. *Journal of Biomedical Optics*.

[37] Lay C. C., Davis M. F., Chen-Bee C. H., Frostig R. D. (2011). Mild sensory stimulation reestablishes cortical function during the acute phase of ischemia. *The Journal of Neuroscience*.

[38] Lay C. C., Davis M. F., Chen-Bee C. H., Frostig R. D. (2010). Mild sensory stimulation completely protects the adult rodent cortex from ischemic stroke. *PLoS ONE*.

[39] Liao L. D., Bandla A., Ling J. M. (2014). Improving neurovascular outcomes with bilateral forepaw stimulation in a rat photothrombotic ischemic stroke model. *Neurophotonics*.

